# Rapid virus-free production of recombinant yellow fever virus envelope protein and its in-depth biophysical analysis

**DOI:** 10.1038/s41598-025-33406-8

**Published:** 2025-12-29

**Authors:** Luca Schelle, Wolfgang Kuttenlochner, Victoria Sanchez, Barbara Steigenberger, Katja Finkl, Marius Schmid, Celine Douat, Einar Halldorsson, Maren Schubert, Sabine Suppmann

**Affiliations:** 1 Fraunhofer Institute for Translational Medicine and Pharmacology ITMP, Immunology, Infection and Pandemic Research IIP, Nonnenwald 2, D-82377 Penzberg, Germany; 2 Max Planck Institute of Biochemistry, Am Klopferspitz 18, D-82152 Martinsried, Germany; 3 ZentriForce Pharma Research GmbH, Carl-Friedrich-Gauß-Ring 5, Überherrn, Heidelberg, D-69124 Germany; 4 Institute for Chemical Epigenetics, Ludwig-Maximilians-Universität, Butenandtstraße 5–13, D-81377 Munich, Germany; 5 FIDA Biosystems Aps, Generatorvej 6 A + B, Søborg, 2860 Denmark; 6 Helmholtz Centre for Infection Research, Inhoffenstraße 7, D-38124 Braunschweig, Germany

**Keywords:** Biochemistry, Biological techniques, Biotechnology, Microbiology, Molecular biology

## Abstract

The aim of this study was to develop a rapid and easy-to-adapt protocol for recombinant production of Orthoflavivirus proteins. We introduced the new-generation pOpiE2 vector for virus-free transient transfection of *Trichopluisa ni* High Five insect cells to produce the envelope protein ectodomain of Yellow Fever Virus Asibi and 17D strains. We achieved high yields in the range of 6–7 mg/L within five working days. In-depth biophysical analysis demonstrated highest protein purity, homogeneity, stability, and affinities to specific antibodies in the low nanomolar range. A combination of peptide mass fingerprint and intact mass spectrometry characterized the copurified precursor chaperone peptide pr and discovered a new posttranslational processing site for the recombinantly produced protein.

**Supplementary Information:**

The online version contains supplementary material available at 10.1038/s41598-025-33406-8.

## Introduction

**Fig. 1:**
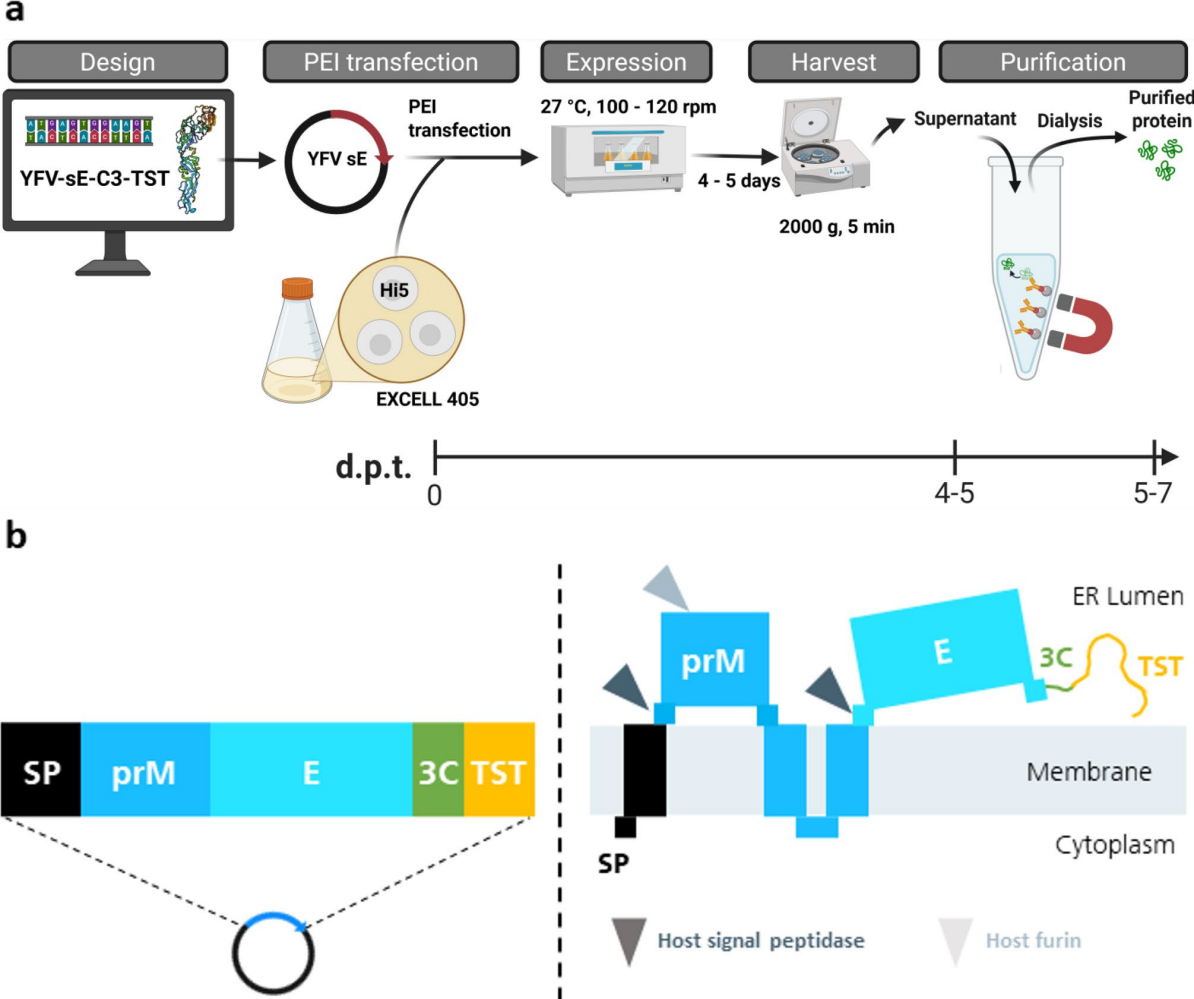
Schematic overview of sE protein production workflow. (**a**) Expression and purification workflow as described under Materials and Methods. Purified protein is available after 5–7 days post transfection (d.p.t.) for small and for large scale productions. This image was generated with Biorender.com. (**b**) The construct design for the expression of sE is depicted as a linear sequence that was cloned into the vector backbone. The orientation of the individual protein parts in the cytoplasm and the endoplasmic reticulum (ER) and the proteolytic sites are indicated with arrows. The prM-E sequences cover amino acids 123–680 of the YFV strains Asibi and 17D, followed by a PreScission (3C) cleavage site and a TwinStrep (TST) tag.

The yellow fever virus (YFV, *Orthoflavivirus flavi*) is a mosquito-borne Orthoflavivirus that is endemic to tropical and subtropical regions of Africa and South America. It causes an acute viral hemorrhagic disease, that leads to symptoms such as fever, chills, muscle pains, and headaches, with a significant number of fatal cases^1^. Although the highly effective YF17D live attenuated vaccine can provide lifelong protection, supply shortages can cause problems in remote areas during outbreaks^2,3^. Moreover, further members of the genus Orthoflavivirus pose geographically distinct public health burdens, especially the mosquito-borne Dengue viruses, Zika virus, Japanese encephalitis virus, West Nile virus and the tick-borne encephalitis virus^4–7^. Orthoflaviviruses are small, enveloped viruses containing a positive-sense single-strand RNA genome encoding one open reading frame processed by host and viral proteases into three structural proteins and seven nonstructural proteins. The structural proteins, namely the capsid protein (C), the membrane-embedded envelope (E) and the pre-membrane protein (prM) form the virion, while the nonstructural proteins are involved in viral replication^8–11^. There is an increasing demand for recombinant Orthoflavivirus proteins for various diagnostic and therapeutic applications. The structural proteins are suitable targets for vaccine development, isolation of neutralizing antibodies, and monitoring the B cell response^12–15^. In addition, the nonstructural protein NS1 is a promising candidate for virus-specific serological assays^16–18^. However, recombinant production of Orthoflavivirus proteins, particularly the membrane-embedded structural envelope protein (E), is a difficult task due to the complex nature of the polyprotein processing steps that involve viral and host proteases^8–11^. Various expression systems have been applied to produce the E protein of Orthoflaviviruses. The prokaryotic host *E. coli* has been successfully exploited to express the E protein or single domains of different Orthoflaviviruses^19–25^. While these protocols have primarily used refolding of individual E domains from inclusion bodies, eukaryotic expression systems have been useful for applications in which preserving the native quaternary structure of the envelope protein is crucial, e.g. for eliciting neutralizing antibody responses. In particular insect cells such as Sf9 from *Spodoptera frugiperda*, High Five from *Trichopluisa ni (T. ni)*, and S2 cells from *Drosophila* have been applied to produce E protein either as soluble ectodomain (sE) or membrane embedded in virus like particles (VLPs) or displayed on baculoviruses^8–45^. The Baculovirus Expression Vector (BEV) System and S2 stable cell lines used in these studies can achieve high expression levels, but have two major disadvantages. Firstly, they are time-consuming, requiring three to four weeks for either baculovirus generation or stable S2 cell line generation^46–48^. Secondly, they require sophisticated protocols and expert knowledge^46–48^. Even though plasmid-based, virus-free transient expression in insect cells had been introduced decades ago as a rapid and easy-to-use alternative, protein yields were very low and not suitable for robust protein production^49–52^. Just recent advances in the expression vector design and protocol have enabled to transiently express proteins in High Five cells from *T. ni* with high efficiency^53–55^. In this study, we exploited this new-generation transient gene expression approach to produce the E protein ectodomain of YFV Asibi and 17D strains as a proof-of-concept project for Orthoflavivirus proteins. We present a fast and easy-to-adapt protocol that yields properly folded, non-aggregated protein within five working days (Fig. 1a). Furthermore, we adhered to protein quality control guidelines^56,57^ and assessed purity, homogeneity, identity, and stability of the purified protein samples. Using in-depth mass spectrometry (MS) analysis, we characterized the copurified pr peptide, which revealed a yet undescribed pre-membrane protein - envelope (prM-E) cleavage site.

## Results

### Protein expression and purification

The soluble envelope ectodomains sE-Asibi and sE-17D were expressed in High Five insect cells using a construct comprising the prM – E (ectodomain) region of the YFV followed by a C-terminal TwinStrep tag (Fig. 1b). These constructs result in the secretion of sE, while M remains membrane-anchored in the cell and the precursor (pr) peptide remains non-covalently bound to sE. The sE – TwinStrep protein is released by a host signal peptidase, whereas the pr peptide is processed by the combination of the host signal peptidase and furin (Fig. 1b). Protein secretion into the culture supernatant was mediated by the vector encoded signal sequence and driven by the constitutive OpiE2 promotor^54^. An initial time course experiment determined optimal harvest time points between four to six days post transfection (data not shown). In addition, furin was coexpressed to overcome a potential limitation in prM-E processing. This did not have a significant impact on protein yields and hence was not used in future experiments.

**Fig. 2:**
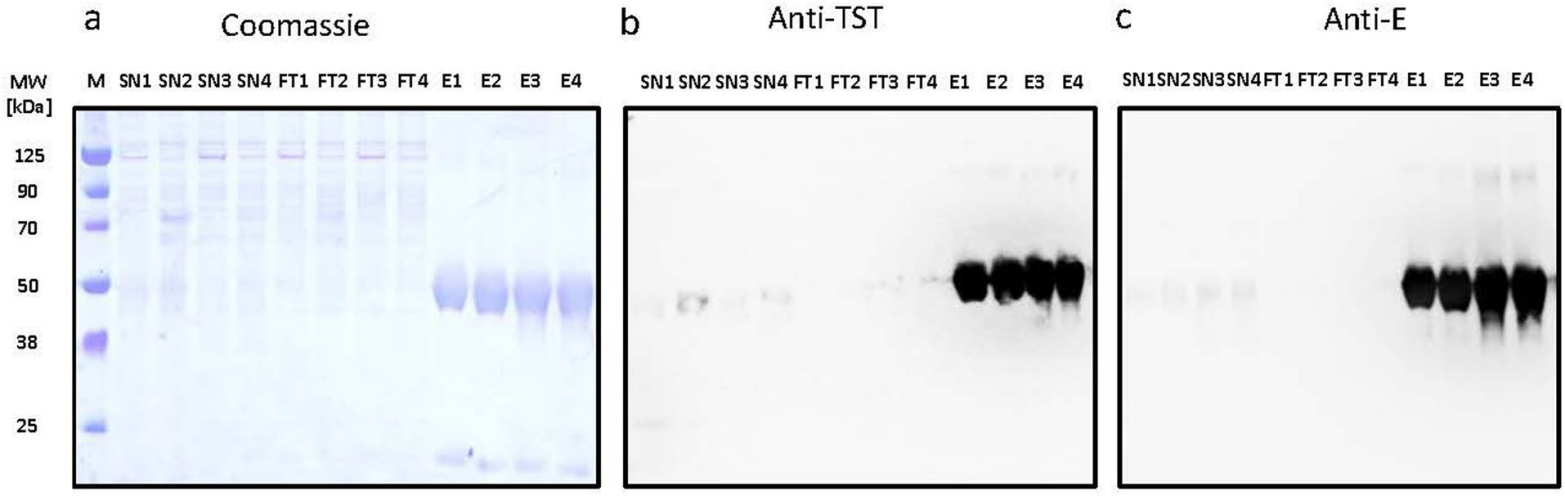
SDS-PAGE analysis of sE-17D and sE-Asibi protein purification. Results of a 20 mL small scale expression experiment harvested and purified 4 days post transfection. 10 µL samples of each purification step were loaded on 4–12% NuPAGE gels for (**a**) direct Coomassie staining; for Western Blot transfers probed with (**b**) StrepTactin-HRP; (**c**) Yellow Fever polyclonal rabbit anti-E (PA5-112024) and goat anti-rabbit IgG (H + L)-HRP secondary antibody (1721019), Bio-Rad Laboratories. M Protein Marker Novex Sharp; SN culture supernatant; FT flow through and E eluate of magnetic StrepTactinXT beads; numbering 1 to 4 corresponds to (1) sE-Asibi; (2) sE-Asibi coexpressed with hs furin; (3) sE-17D (4) sE-17D coexpressed with hs furin; coexpression was performed using a plasmid ratio of sE: hs furin at 1:4. Images were cropped vertically to remove non-related content. Full images are displayed in Supplementary Fig. S8.

**Fig. 3:**
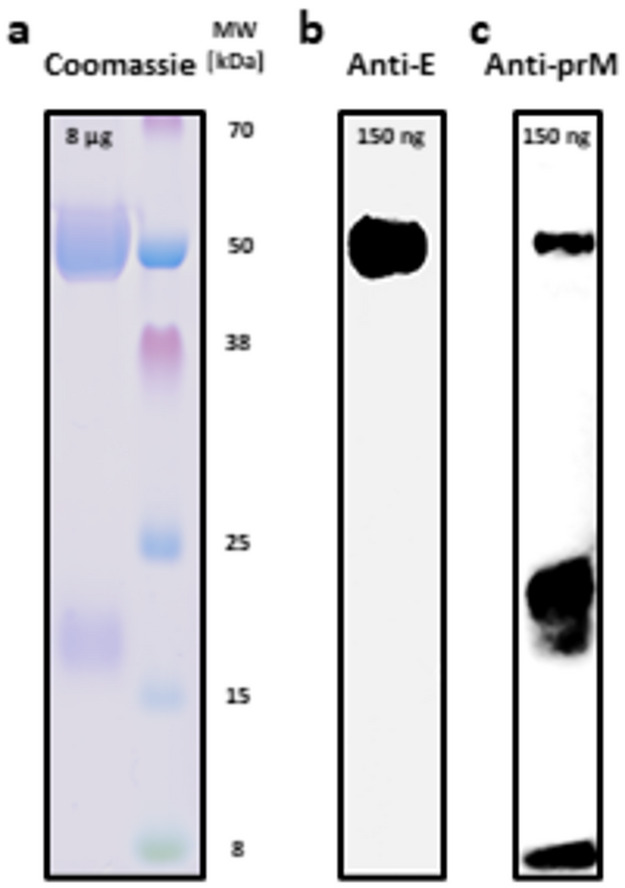
SDS-PAGE analysis of sE-Asibi. Indicated amounts of purified sE-Asibi were loaded on 4–12% denaturating NuPAGE gels for (**a**) direct Coomassie staining; for Western Blot transfers probed with (**b**) polyclonal rabbit anti-E; (**c**) polyclonal rabbit anti-prM detected with HRP-labelled anti-Rabbit IgG secondary antibody. Gel electrophoresis time was shortened for optimal coverage of pr peptide. Images were cropped vertically to remove non-related content. Full images are displayed in Supplementary Fig. S8.

Single-step affinity purification of the culture supernatant after four days of transient expression revealed one major protein band at 50 kDa, which was detected at similar intensities for sE-Asibi and sE-17D by Coomassie staining (Fig. 2a) and by StrepTag (Fig. 2b) and envelope protein specific antibodies (Fig. 2c). Using a higher sE-Asibi protein load, the faint small protein band was identified as the co-purified pr peptide that tightly binds to sE (Fig. 3a) confirmed by specific Western blot detection using envelope (Fig. 3b) and prM (Fig. 3c) antibodies. In agreement with previous reports, the pr peptide with an expected size of 9 kDa migrates aberrantly around 18 kDa^58,59^. The strength of binding of pr to sE protein is corroborated by the fact, that a minor part of pr comigrates with sE in a denaturating SDS gel even after 5 min 95 °C sample treatment in SDS Laemmli buffer. Final yield after dialysis of pooled fractions from the StrepTactinXT resin into PBS buffer was 6 and 7 mg per L culture for sE-17D and sE-Asibi, respectively (Fig. 1S). A final purity of 95–98% (E + pr) was reproducibly achieved in eight different purification batches, from production scales of 20 mL to 2 L.

### Quality control

**Fig. 4:**
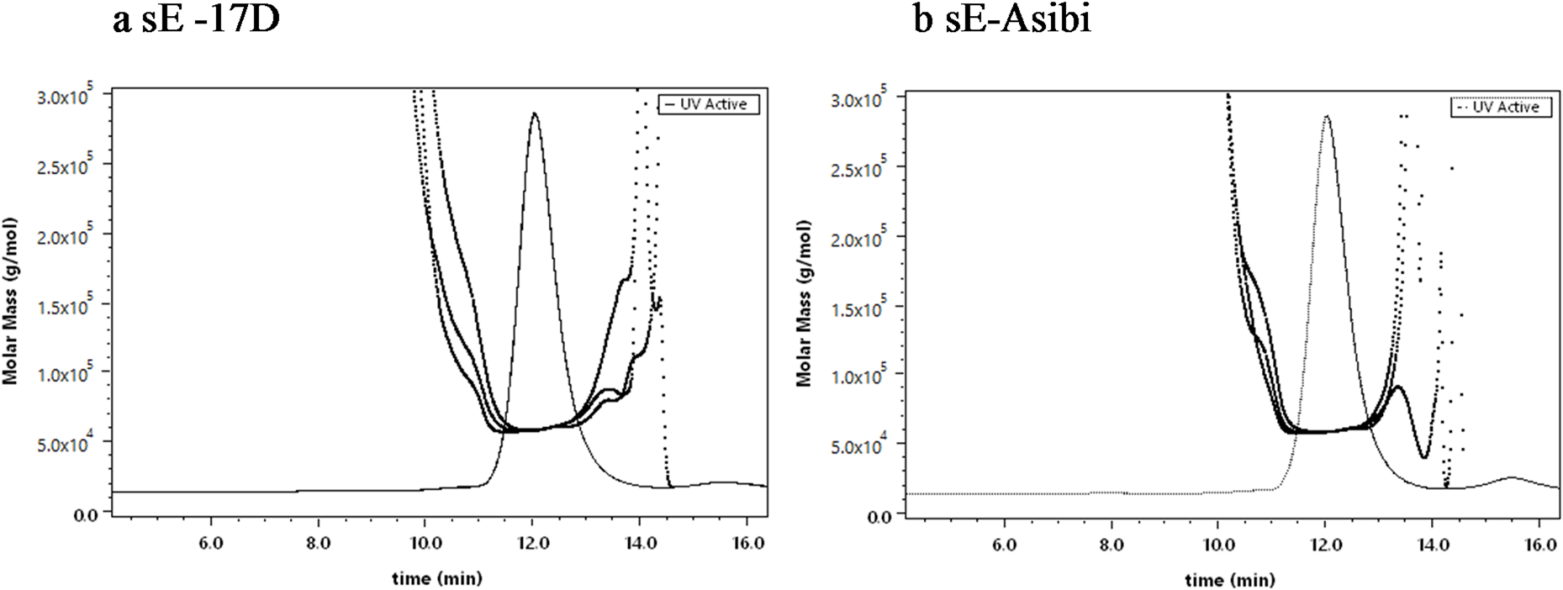
SEC-MALS of purified sE-17D and sE-Asibi proteins. 25 µL protein samples of **a**) sE-17D (1 mg/mL) and **b**) sE-Asibi (1 mg/mL) were loaded on an AdvanceBio SEC 300 Å HPLC column and separated at a flow rate of 0.25 mL/min with 150 mM sodium phosphate at pH 7.0. The UV trace recorded at 280 nm shows one major peak at 12 min elution time. MALS analysis of the peaks (bold lines) and calculation based on the first order Zimm Plot resulted in molecular weights of 58–59 kDa for sE-17D and sE-Asibi.

In a next step, the oligomerisation status as well as aggregation content of the sE-Asibi and sE-17D protein samples were determined by Size Exclusion Chromatography coupled to a Multi-Angle Light Scattering detector (SEC-MALS). For both purified protein samples, a single peak was detected and its molecular mass determined at 58–59 kDa for the entire peak elution time (Fig. 4). Given a theoretical mass of 47.2 kDa for sE and a 9.8 kDa pr peptide harboring two N-glycan modifications, this size corresponds to a monomeric sE-pr protein-peptide complex.

Because high molecular weight aggregates could have been removed on the prefilter or on the matrix of the size exclusion column, we applied Dynamic Light Scattering (DLS) and Taylor dispersion (TD) as complementary approaches. In both methods, the entire sample is measured in batch without any potential removal of aggregates. DLS analysis confirmed, that sE-Asibi and sE-17D protein samples are highly monodisperse without any aggregate content (Fig. 2S; Table 1S). In addition, DLS and TD analysis showed, that monodispersity is maintained after a short freeze-thaw cycle at -80 °C and also after a four week − 80 °C storage (Fig. 3S; Table 1S).

**Fig. 5:**
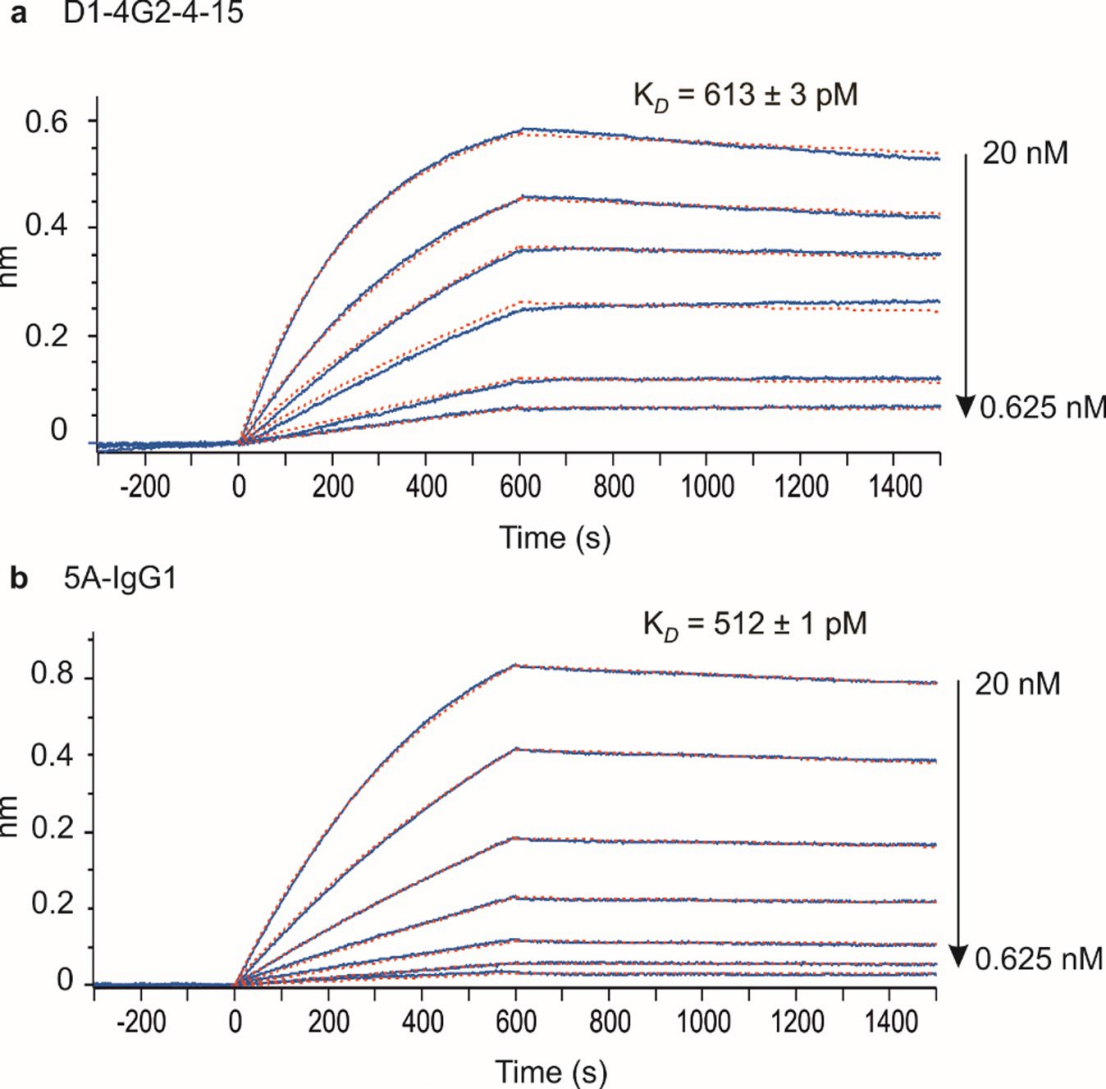
Affinity measurement by Biolayer Inferometry. BLI kinetic sensorgrams (black curves) and curve fittings (dash red curves) to determine the K_*D*_ values of the binding of sE-Asibi protein to (**a**) the mouse antibody D1-4G2-4-15 captured on AMC2 biosensors and (**b**) the human antibody 5 A-IgG1 captured on AHC2 biosensors. The reported K_*D*_ values are the mean of *N* = 3 experimental replicates (see supporting information for experimental details Fig. 4S).

Having passed these quality criteria, namely purity, homogeneity, and stability, we tested if the sE protein adopts a functional conformation with its epitopes displayed properly. Therefore, we measured specific binding affinities of sE to previously described D1-4G2-4-15 (4G2) and 5 A antibodies. 4G2 is a mouse recombinant monoclonal antibody binding to the fusion loop (FL) of various Orthoflaviviruses^60^, while 5 A is a human YFV specific monoclonal antibody and recognizes an epitope in DII and DI in pre- and postfusion state which was suggested to be at the pr binding site^21^. Biolayer Inferometry (BLI) analysis determined K_D_ values in the subnanomolar range for 4G2 (613 pM), and 5 A (512 pM), (Figs. 4S and 5) with sE-Asibi, thus proving a proper three-dimensional structure of the binding domains DI and DII, which also indicates correct post-translational processing and overall folding using the described production method.

### In-depth analysis of the sE-pr protein-peptide complex

An additional and indispensable quality control of the purified protein sample is Mass Spectrometry (MS). It proofs the identity of the sample and can detect small truncations, amino acid exchanges, nonproper signal peptide cleavage, or else^56^.

**Fig. 6:**
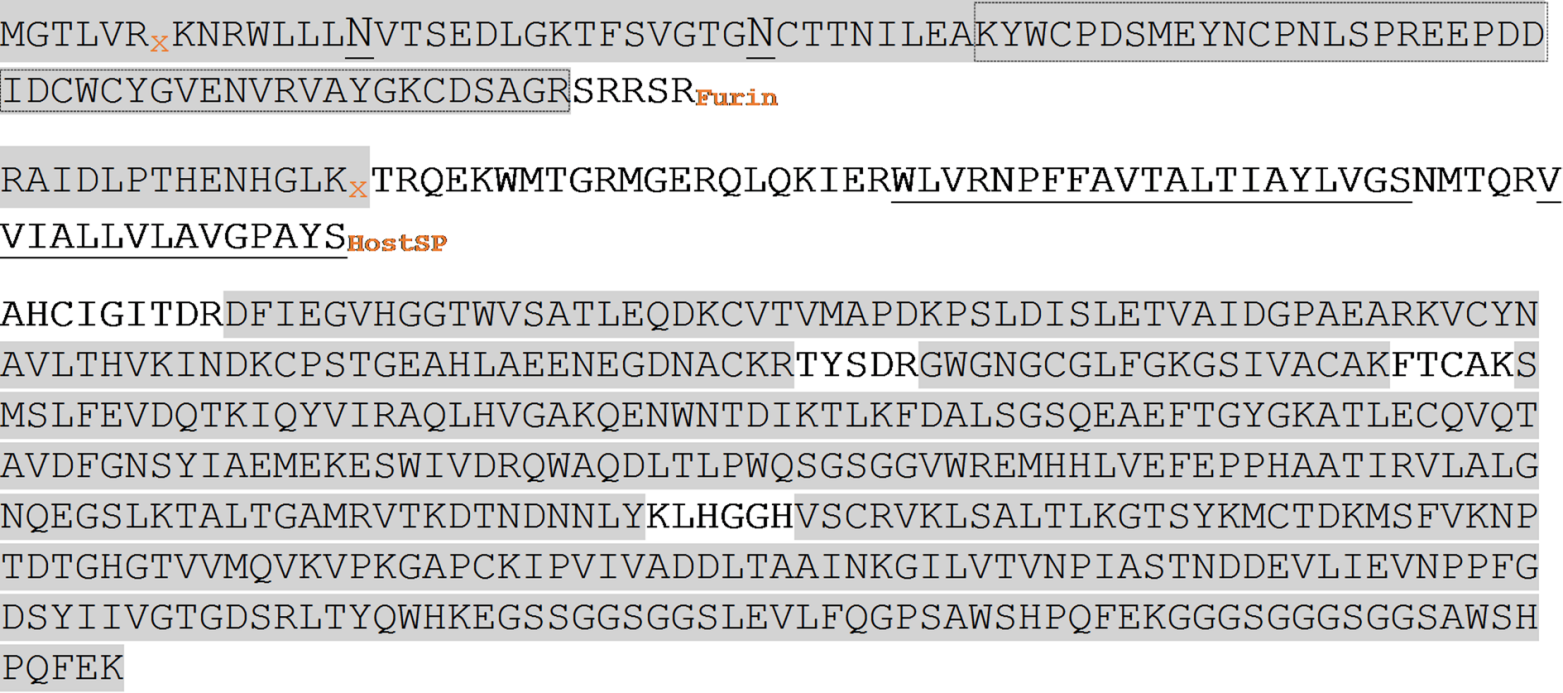
Tryptic peptide mass fingerprint coverage of the sE-Asibi construct. Peptide coverage after deglycosylation and tryptic digest of the purified fusion protein Asibi prM-E-3 C-TST; the theoretical fragments generated by host proteases are displayed in three separate rows. *(i)* pr peptide ending with a furin cleavage site; the two N-glycosylation sites are underlined; *(ii)* small envelope protein M ending with the host signal peptidase cleavage site; this fragment is membrane bound with the two transmembrane stretches underlined and not expected to be copurified with soluble envelope protein; *(iii)* envelope protein E with C-terminal GSSGGSGG linker, PreScission site (LEVKQGP) and TwinStrep tag (SAWSH….PQFEK); peptides identified with high significance are highlighted by grey boxes; Grey boxes framed by a dotted line highlight peptides that were additionally identified by in-gel tryptic digest of the SDS-PAGE separated peptide band (Fig. 3a). x depicts potential cleavage sites of the second pr peptide. Peptide coverage was almost identical for 17D prM-E-3 C-TST (Fig. 5S).

First, we confirmed the identities of the sE proteins by performing tryptic digestion followed by peptide mass fingerprint analysis. This revealed the expected peptide masses for sE-Asibi (Fig. 6) and sE-17D (Fig. 5S), respectively, as well as peptides that originate from processing of prM that are non-covalently bound to sE^8,9,10,11,59,60^. 

In the peptide mass fingerprinting data, we consistently observed extensive coverage of a tryptic peptide RAIDLPTHENHGLK. Multiple peptide-spectrum matches were identified for this peptide and its modified variants indicating that this region was highly represented in the digest. The repeated detection of this peptide suggested the presence of a stable fragment around this sequence. The peptide spectrum matches and intensity for this peptide was similar high as for N-terminal peptides. Consequently, the presence of the co-purified RAIDLPTHENHGLK peptide fragment indicated, that processing of the prM-E-3 C-TST fusion protein in High Five insect cells might involve a so far undescribed non-furin proteolytic cleavage site.

To derive the processing position of the peptides, we performed intact mass analysis. The sE-pr protein-peptide complex was separated by liquid chromatography (LC) on a C18 reverse phase column and the masses of the individual eluting peaks were determined by online MS. As a result, we determined LC peaks corresponding to the soluble E-3 C-TST protein (41.7 kDa, Fig. 6S; Table 1) and the pr peptide with glycan modifications showing a diffuse size distribution around 12 kDa (data not shown). Hence, we deglycosylated the sample with PNGaseF, which resolved the single LC peptide peak into three separate species (Fig. 6S; Table 1): One of them being a result of incomplete deglycosylation with its diffuse size distribution. The second peak with a mass of 9.95 kDa corresponds to the expected size of the canonical furin cleavage pr peptide^58,59^. The third peak has 11.14 kDa in size which must include amino acids past the furin cleavage site. This unexpected fragment may be a cleavage product generated at the position x-x depicted in Fig. 6.

**Table 1:** Molecular masses identified in purified and deglycosylated sE-Asibi protein.

Fragment	Mass measured [Da]	Theoretical Mass [Da]	Mass difference [Da]
peptide pr Furin	9953.23	9871.11	+ 82.12
2nd peptide x-x	11138.84	11039.33	+ 99.51
E-3 C-TST	47100.60	47241.20	− 140.60

Individual fragments of the purified sE-Asibi protein sample were separated with HPLC and the molecular masses of three individual peaks determined at high accuracy (see Fig. 6S). Envelope E-3 C-TST and pr peptide after furin cleavage show minor deviations to the expected fragment masses. The molecular mass of a 2nd peptide most closely fits to a potential x-x fragment displayed in Fig. 6.

To further test our hypothesis, we conducted native MS analysis of the deglycosylated complex without prior separation of the sE protein and pr peptide components. This experiment confirmed the existence of two different sE-pr complexes with total molecular masses that are identical to the sum of sE with either pr1 (9.95 kDa) or pr2 (11.14 kDa) (Table 2; Fig. 7S).

**Table 2:** Native masses identified for sE-Asibi protein-peptide complexes.

Fragment	Deglycosylation	Mass measured [Da]	Corresponding fragment masses
E + pr1 / pr2	-	59,330	47.100.60 + ca. 12.000
E + pr1	+	57,050	47100.60 + 9953.23 = 57053.83
E + pr2	+	58,240	47100.60 + 11138.84 = 58238.84

Molecular masses were measured for protein-peptide complexes without prior fragment separation (see Fig. 7S). Protein-peptide complex sizes correlate with the combination of individual fragment masses detected in LC-MS (see Fig. 6S; Table 1).

In summary, using in-depth MS analysis we were able to identify two different pr peptides that non-covalently bind to sE during purification.

## Discussion

The increasing prevalence of infections with Orthoflaviviruses creates an urgent need for rapid, sensitive, and specific diagnostic tests and therapeutic interventions. Although Reverse Transcription Polymerase Chain Reaction (RT-PCR) and virus isolation from the blood of infected patients allow a reliable diagnosis, they are limited to the short viremic phase of an acute infection^53,61,62^. In contrast, immunological assays such as enzyme-linked immunosorbent assays (ELISA) or lateral flow assays can detect antibodies directed against the Orthoflavivirus antigens E and nonstructural protein NS1 up to several months (IgM) or years (IgG) post-infection^16,17,61,64^. However, serological diagnosis of flavivirus-associated diseases is hindered by a high degree of cross-reactivity against E antigens from different flaviviruses and by limited sensitivity for NS1^61,65,66^. To enable and facilitate broader studies that address these problems in more detail, we have developed a rapid, robust, and easy-to-use protocol for Orthoflavivirus protein purification using the YFV E protein as a first proof-of-concept. Applying virus-free plasmid-based expression in High Five insect cells in combination with a single-step TwinStrep tag affinity purification, we produced sE from Asibi and 17D YFV in a 6–7 mg/L range within five working days. Highest protein quality is essential for the success of downstream applications. In an effort to improve the reliability of published data overall, the ARBRE-MOBIEU and P4EU networks have proposed minimum quality control standards^56,57^. We have thoroughly followed these guidelines and demonstrated a high degree of purity, monodispersity, and stability of the purified proteins. In addition, we showed that sE-Asibi binds to 4G2 and 5 A specific antibodies with high affinities in the subnanomolar range concluding the presence of an intact fusion loop (4G2) and an DII-DI epitope which is present in the mature and post-fusion state in the virus. This finding is in agreement with previous studies, that had used a similar expression construct in S2 insect cells and has implications for further downstream applications^58,59^. Another consistent finding with these studies is the fact, that we co-purified soluble sE protein in complex with a non-covalently bound precursor peptide pr, as shown by SDS-PAGE and Western Blot analysis. To elucidate the nature of the pr peptide in more detail, we applied a combination of three complementary MS methods. As expected, we identified a peptide pr1 of 9.95 kDa which corresponds to the described cleavage product of the prM-E precursor (Fig. 1b).

In addition to the expected pr1 peptide, we identified another non-canonical pr2 peptide (11.14 kDa) in the purified protein sample, that must have emerged from an alternative posttranslational cleavage event downstream of the furin site. The molecular mass of pr2 most closely fits to a potential x-x fragment displayed in Fig. 6. This pr2 peptide contains part of the membrane bound M protein and was not expected to be co-purified with the sE ectodomain^8–11,58,59^. A molar ratio of sE: pr of 1:1 was determined by SEC-MALS and the existence of two different pr peptides can only be explained by a mixture of two distinct protein-peptide complexes. Native MS analysis has indeed confirmed the existence of two different sE: pr1 and sE: pr2 protein-peptide complexes. The ratio of the two complexes cannot be quantified by MS methods and will need more elaborate efforts which are out of the scope of this study. To our knowledge, the pr peptide in similar sE-pr co-purifications has never been analyzed by MS and attempts to elucidate the entire structure of pr in sE-pr crystals have failed so far^58,59^. It remains to be shown, if this hitherto undescribed cleavage site is also utilized in infected cells during native YFV maturation. Experiments with West Nile virus have shown that the infectivity of immature virions is not affected, when cells are treated with a potent furin inhibitor, indicating the existence of a non-furin cleavage site^68,69^. Thus, our findings open up the floor for further studies on glycoprotein processing by insect specific proteases and its implications on viral infection pathways^70,71^.

The identification of a second pr peptide highlights the importance of MS analysis to provide a full view of protein samples, which can have a major impact on downstream applications such as serological assays. A combination of complementary biophysical characterization methods and three different MS experimental set-ups were necessary to reveal the existence of a mixture of two different E-pr protein-peptide complexes in the YFV sE-Asibi sample.

In summary, we have expanded the existing portfolio of expression methods for Orthoflavivirus proteins with a next-generation, plasmid-based transient expression protocol. Unlike existing procedures, this protocol combines speed, yield, and quality in an unprecedented way. Its simplicity enables non-expert users to generate significant protein yields within five working days. In addition, plasmid-based expression in High Five cells is as a powerful tool for the production of VLPs^72,73^. As proof-of-concept, we have developed the protocol for YFV sE protein and further studies will likely reveal its wide applicability to produce other complex proteins.

## Materials and methods

### Construct design

prM-E sequences (amino acid 123–680) of YFV strain Asibi (UniProt Acc. no Q6DV88) and 17D (UniProt Acc. no P03314) were fused to a C-terminal GSSGGSGGS linker followed by a PreScission cleavage site (3 C) for potential tag removal and a TwinStrep tag (TST). The fusion constructs prM-E-3 C-TST were ordered at GeneArt, (ThermoFisher, Regensburg, Germany) and cloned via *Nco*I/*Not*I into pOpiE2 in frame with the vector encoded signal sequence. The cloning step adds two non-native amino acids (MG) to the N-terminus of prM-E.

### Recombinant protein expression in insect cells

High Five cells (BNI-TN-5B1-4; Thermo Fisher Scientific) were cultivated in EX-CELL 405 serum-free medium (Sigma Aldrich) at 27 °C and 100–120 rpm depending on flask size at a cell density between 0.3 and 5.5 × 10^6^ cells/mL. Transfection was performed as described previously^21^. Briefly, cells from a log-phase culture were centrifuged for 4 min at 180 × g. The cell pellet was adjusted to 4 × 10^6^ cells/mL in fresh EX-CELL 405 medium in 25% of the desired transfection volume. 1 µg plasmid DNA and 4 µL transfection grade linear Polyethylenimine Hydrochloride (MW 40,000; 1 mg/mL stock solution; Polysciences Europe GmbH) per 1 × 10^6^ cells were added separately directly to the cell suspension. For coexpression of furin with sE, the respective plasmids were mixed in a 1:5 ratio of the total amount of DNA. About 6–21 h post transfection, 3-fold of the initial volume of fresh medium was added to adjust the cell number to ~ 1 × 10^6^ cells/mL. Another feed with 100% transfection volume was performed 48 h post transfection. The cell culture supernatant was harvested 4–5 days post transfection.

### Protein purification

For small scale expression tests, 20 mL culture supernatants were harvested by centrifugation at 840 x g for 5 min at 4 °C, incubated with 40 µL BioLock (IBA LifeSciences, Göttingen) for 15 min on ice to block medium biotin content and subsequently loaded on 30 µL MagStrep Strep-Tactin beads (IBA LifeSciences, Göttingen) preequilibrated with phosphate buffered saline (PBS). Binding was allowed for 2 h at 4 °C. Beads were collected on a 6-Tube Magnetic Separation Rack (NEB), washed three times in 1.5 mL PBS each and eluted with with PBS + 50 mM biotin (Sigma Aldrich) in 50 µl fractions with 10 min incubation on ice. Protein concentration was determined using the Pierce™ Bradford Plus Protein Assay Kit (ThermoFisher Scientific) and measured on a NanoPhotometer NP80 (IMPLEN, Munich). Protein containing fractions were pooled and dialyzed overnight against PBS buffer using Pur-A-Lyzer Mini 6000 devices (Sigma Aldrich). UV spectra were recorded on the NP80. For large scale purification, 1 L High Five cell culture supernatant was harvested 5 days post-transfection by centrifugation at 840 x g for 10 min at 4 °C, incubated with 2 mL BioLock (IBA LifeSciences, Göttingen) for 20 min on ice, sterile filtered and subsequently loaded on an Econo-Pac Chromatography Column (Bio-Rad Laboratories, Germany) filled with 2 mL Strep-Tactin^®^XT 4Flow^®^ resin (IBA Göttingen, Germany) preequilibrated with PBS. Sample load was achieved by gravity using the Wet FRED loading device (IBA Göttingen, Germany) over night at 4° C. Next morning, beads were washed in 20 mL PBS; protein was subsequently eluted with 4 mL PBS + 50 mM biotin (Sigma Aldrich) in 0.5 mL fractions. Protein containing fractions were pooled and dialyzed overnight against PBS buffer using D-Tube Dialyzer Maxi devices, MWCO 6-8kD (Sigma Aldrich). Protein aliquots were stored at -80 °C.

### SDS-PAGE and Immunoblot analysis

Proteins were separated on NuPAGE 4–12%, Bis-Tris (ThermoFisher Scientific) in MOPS running buffer and either stained with Coomassie R-250 (Imperial Protein Stain, ThermoFisher Scientific) or blotted semi-dry onto NC-45 nitrocellulose membrane (SERVA Electrophoresis, Heidelberg) using the Trans-Blot Turbo System (Bio-Rad Laboratories GmbH, Germany). The membrane was blocked with PBS containing 3% BSA and 0.05% Tween 20 for 1 h at RT. For TwinStrep tag fusion protein detection, HRP-labelled StrepTactin (2-1502-001, IBA LifeSciences, Göttingen) was used according to the manufacturer instructions. For specific detection of Yellow Fever Virus proteins, polyclonal rabbit anti-E and anti-prM antibodies (PA5-112024 and PA5-112019 ThermoFisher Scientific) were used. After 3-fold washes with PBS + 0.05% Tween 20, the blot was probed with Goat Anti-Rabbit IgG (H + L)-HRP Conjugate secondary antibody (1721019, Bio-Rad Laboratories, Germany). Staining was performed with Clarity Western ECL Substrate (Bio-Rad Laboratories, Germany) followed by visualization with the Odyssey M Imager (Li-COR, USA). Intensity of Coomassie-stained bands was quantified based on near red fluorescence at 700 nm and analyzed with the Empiria Studio 3.2 Software.

### SEC-MALS

Size exclusion was performed on an Agilent 1260 Infinity II HPLC system. The proteins were separated on an Agilent AdvanceBio SEC 300 Å column (2.7 μm, 4.6 × 300 mm) at 30 °C in a Shimadzu CTO-20AC column oven. The HPLC system was serially connected to an Agilent 1260 Infinity II DAD UV detector, a Wyatt Dawn Heleos II MALS detector and a Wyatt OptiLab T-rEX RI detector. SEC-MALS measurements were performed in 150 mM sodium phosphate pH 7.0 running buffer at a flowrate of 0.25 mL/min with a sample injection volume of 25 µl. The instrument was operated by the software Vision (v 3.2.0.67), and Chromelion 7, respectively. Data collection and evaluation were controlled by the Wyatt ASTRA software (v 8.2.2.101). A first order Zimm Plot was used to calculate the molecular weight. An extinction coefficient of 1.609 mL/mg cm and a refractive index increment of 0.0185 mL/g were applied.

### Mass spectrometry

For deglycosylation, 20 µL of purified sE Asibi solution in PBS (1.89 mg/mL) was incubated with 3 µL of PNGase F (New England Biolabs) at 37 °C overnight. For peptide mass fingerprint analysis, the deglycosylated sample was incubated for 20 min at 37 °C with 20 µL SDC buffer containing 1% sodium deoxycholate, 40 mM 2-chloroacetamide (Sigma-Aldrich), 10 mM tris-2-carboxyethylphosphin hydrochloride (TCEP, Thermo Fisher Scientific) in 100 mM Tris, pH 8.0. Subsequently, 40 µL of MilliQ water and 0.5 µg trypsin (Promega) was added for tryptic digestion overnight at 37 °C. The solution was acidified with trifluoroacetic acid (TFA; Merck) to a final concentration of 1%. Any precipitated SDC was removed via centrifugation, and the peptide mixture was loaded on Evotips (Evotip Pure, Evosep). For LC-MS/MS Evotips were eluted onto a 15-cm column (PepSep C18, 15 cm x 15 cm, 1.5 μm, Bruker Daltonics) using the Evosep One HPLC system. The column was heated to 50 °C, and peptides were separated using the 30 samples per day (SPD) method. Subsequent data acquisition on the timsTOF Pro mass spectrometer was carried out as reported previously^42^. Raw data were processed using the MaxQuant computational platform (v 2.2.2.0) with standard settings applied. Briefly, the peak list was searched against a fasta file containing the sequence of prM-E-3 C-TST of YFV Asibi and background proteins with an allowed precursor mass deviation of 4.5 ppm and an allowed fragment mass deviation of 20 ppm. Cysteine carbamidomethylation was set as static modification, and methionine oxidation and N-terminal acetylation as variable modifications. For LC-MS measurement of the intact masses, 6 µL of the deglycosylated sE protein sample was injected on a C4 Phenomenex AerisTM 200Å column (3.6 μm WIDEPORE 100 mm x 2.1 mm). Chromatography was performed at a flow rate of 250 µL/min at 20 °C using an Agilent 1100 HPLC system coupled to a microTOF mass spectrometer (Bruker Daltonik). UV detection was performed at 214 nm. The eluents consisted of buffer A (0.05% TFA in H2O, pH 2.0) and buffer B (0.05% TFA in ACN, pH 2.0). The microTOF mass spectrometer was operated in positive ion mode, covering a mass range of 800–3000 m/z. MS1 scans were recorded. Data processing was conducted using CompassTM data analysis software (Bruker Daltonik). Deconvolution performed using the maximum entropy algorithm and an instrument resolving power of 10.000. For native MS of protein-peptide complexes, an online buffer exchange coupled to native MS was performed using a Thermo Scientific Vanquish Flex UHPLC System equipped with two pumps coupled to a Orbitrap Exploris 480 mass spectrometer (Thermo Scientific). The column for online buffer exchange was a Thermo Scientific NativePac OBE-1 s column for online sample preparation. Online-buffer exchange was proceeded at a flow rate of 100 µL/min and the mobile phase was 200 mM ammonium acetate buffer. Buffer-exchanged proteins eluted first and were directed to the MS. Subsequent non-volatile salts eluted later and were diverted to waste by a six-port valve using a separate pump. The whole LC-MS run lasted for 3 min. Native MS analyses were performed on an Orbitrap Exploris 480 mass spectrometer in intact protein mode. HESI source parameters were set as follows: sheath gas flow rate, 15 arbitrary units (AU); auxiliary gas flow rate, 10 AU; sweep gas flow rate, 0 AU; spray voltage, 3.20 kV; capillary temperature, 280 °C; and auxiliary gas heater temperature, 100 °C. The mass spectrometer was operated in positive ion mode and MS1 scans were acquired from m/z 1000 to 8000 at a resolution of 15,000. Source fragmentation was set to 60–135 V respectively. The automatic gain control (AGC) target value was set to 300% for MS1. Data analysis was performed using UniDec. Deconvolution settings involved a mass range 1–8 kDa, a charge range of 1–50, and the masses were sampled every 10 Da.

### Biolayer interferometry affinity measurement

Octet^®^ R8 (Sartorius) was used for mouse D1-4G2-4-15 IgG2a kappa (NBP2-52709, Novus Biologics) and human 5 A-IgG1^30^ (produced in-house in HEK293 cells) binding interaction studies. Samples were dispensed in black microplate 96-well plates (Greiner, Germany). The kinetic buffer (KB) used for baseline, capture and kinetic steps was composed of (PBS) supplemented with 0.02% Tween 20, 0.05% sodium azide, and 0.1 mg/mL bovine serum albumin (BSA). According to the manufacturer instructions, the well volume for each step was 200 µL and plate shaking set to 1000 rpm all along the kinetic experiment. All the kinetic assays were performed at 30 °C. Prior to each kinetic assay, anti-mouse IgG Fc capture (AMC2) or anti-human IgG Fc capture (AHC2) biosensor tips (Sartorius) were pre-conditioned in 200 µL of KB in the instrument for at least 10 min at 30 °C. Equilibration of the biosensors for 300 s is followed by the capture of eight biosensor tips with D1-4G2-4-15 (AMC2) or 5 A-IgG1 (AHC2) at 10 µg/mL concentration for 50 s. After a sensor washing for 300 s, the baseline is recorded followed by the association step with a serial twofold dilution of sE protein analyte from 20 nM to 0.125 nM 600 s and the dissociation step for 900 s by dipping the sensors in the same well column where the baseline was performed. The last column biosensor was use to reference the baseline during the reprocessing of the kinetic experiment (no analyte). All measurements were performed in triplicate. The absence of non-specific binding was also confirmed by recording the association/dissociation step on sensor, which has not been captured with mouse D1-4G2-4-15 or human 5 A-IgG1 antibody.

## Supplementary Information

Below is the link to the electronic supplementary material.

Supplementary Material 1

## Data Availability

The datasets generated and/or analysed during the current study are available in the supporting information and UniProt (IDs P03314 for YFV-17D, Q6DV88 for YFV-Asibi, https://www.uniprot.org/uniprotkb/P03314/entry; https://www.uniprot.org/uniprotkb/Q6DV88/entry.
