# Supplementary material for: Rapid virus-free production of recombinant yellow fever virus envelope protein and its in-depth biophysical analysis
Source: Sci Rep. 2025 Dec 29;15:45666. doi: 10.1038/s41598-025-33406-8 (PMC12753717; doi:10.1038/s41598-025-33406-8)
Supplement: Supplementary file 1 — Supplementary Material 1 [file 41598_2025_33406_MOESM1_ESM.docx]

Rapid virus-free production of recombinant yellow fever virus envelope protein and its in-depth biophysical analysis

Supplementary figures and tables

Fig 1S: UV spectrum of purified sE-Asibi and sE-17D proteins


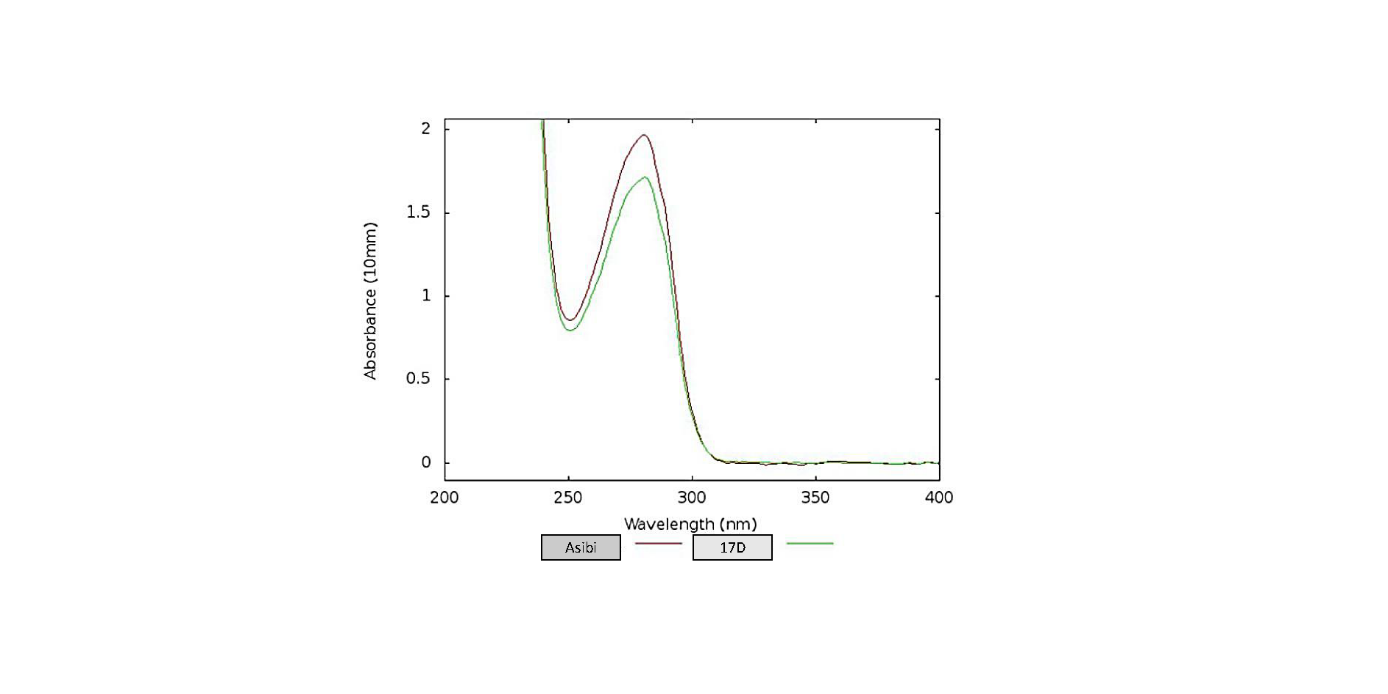


Fig 1S: UV spectra of purified protein samples (Fig. 2 main manuscript) were recorded on the IMPLEM NP80 nanophotometer at 10 mm. Absorption maxima at 280 nm indicate absence of nucleic acids. Protein concentration derived from the sE extinction coefficients was 1.4 mg/mL and 1.2 mg/mL for sE-Asibi and sE-17D, respectively.

Fig. 2S: Dynamic light scattering analysis of protein samples directly after purification

a sE -17D b sE-Asibi


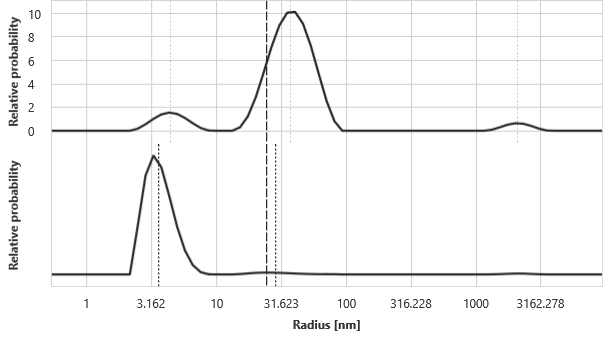

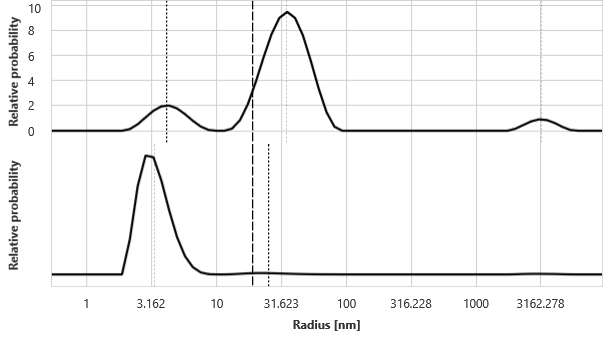


Volume Distribution

Intensity Distribution

Volume Distribution

Intensity Distribution

Fig. 2S: Dynamic light scattering (DLS) results of a) sE-17D (1.4 mg/mL) and b) sE-Asibi (1.2 mg/mL) protein samples measured on a Prometheus Panta instrument (Nanotemper, Germany). Unfiltered protein samples (see UV spectrum in Fig. 1S) were transferred to standard capillaries (Nanotemper, Germany) and loaded onto the instrument sample tray. Measurements were carried out at 15 °C. Autocorrelation functions were fitted using both the size distribution analysis and the cumulant analysis method. The intensity distribution is based on the raw scattering data. As the scattering intensity of a particle is proportional to its radius r^6^, larger particles are overrepresented in the intensity distribution. To correct for this over-representation, intensity distribution is converted to volume distribution using Mie theory and the refractive index of the sample. This conversion implies the assumptions that *i)* a given intensity distribution is correct; *ii)* all particles are spherical and *iii)* have the same refractive index. Therefore, converted values are only reliable in combination with complementary methods such as Taylor Dispersion (Fig 3S.) and SEC-MALS (main manuscript Fig. 3) and are then useful for batch-to-batch comparisons (Table 1).

Table 1S: Protein batch-to-batch comparison by dynamic light scattering

| Sample | Peak 1 rH [nm] | Peak 1 PDI | Peak 2 rH [nm] | Peak 2 PDI |
| --- | --- | --- | --- | --- |
| sE-17D purified | 4.39 | 0.08 | 37.25 | 0.19 |
| sE-Asibi purified | 4.20 | 0.10 | 34.32 | 0.17 |
| sE-17D freeze thaw -80 °C | 4.32 | 0.08 | 34.97 | 0.14 |
| sE-Asibi freeze thaw -80 °C | 4.25 | 0.07 | 32.93 | 0.15 |
| sE-Asibi 4-weeks storage at -80 °C | 4.16 | 0.09 | 28.49 | 0.20 |

Table 1S: Dynamic light scattering was performed as described for Fig. 2S. The size distribution analysis shows hydrodynamic radius (rH) and Polydispersity Index (PDI). 17D- and sE-Asibi protein samples were measured directly after purification (see UV spectrum in Fig 1S.), after one freeze thaw cycle at -80°C, and for sE-Asibi after a 4-weeks storage at -80 °C; in parallel a Taylor-gram was recorded for the same sE-Asibi batch (Fig. 3S). The PDI is a measure for heterogeneity of the particles size distribution; according to the Panta instrument specifications PDI < 0.1 is highly monodisperse; 0.1 - 0.2 is monodisperse and > 0.2 is polydisperse.

Fig 3S: Flow-induced dispersion analysis of sE-Asibi after storage and freeze-thaw
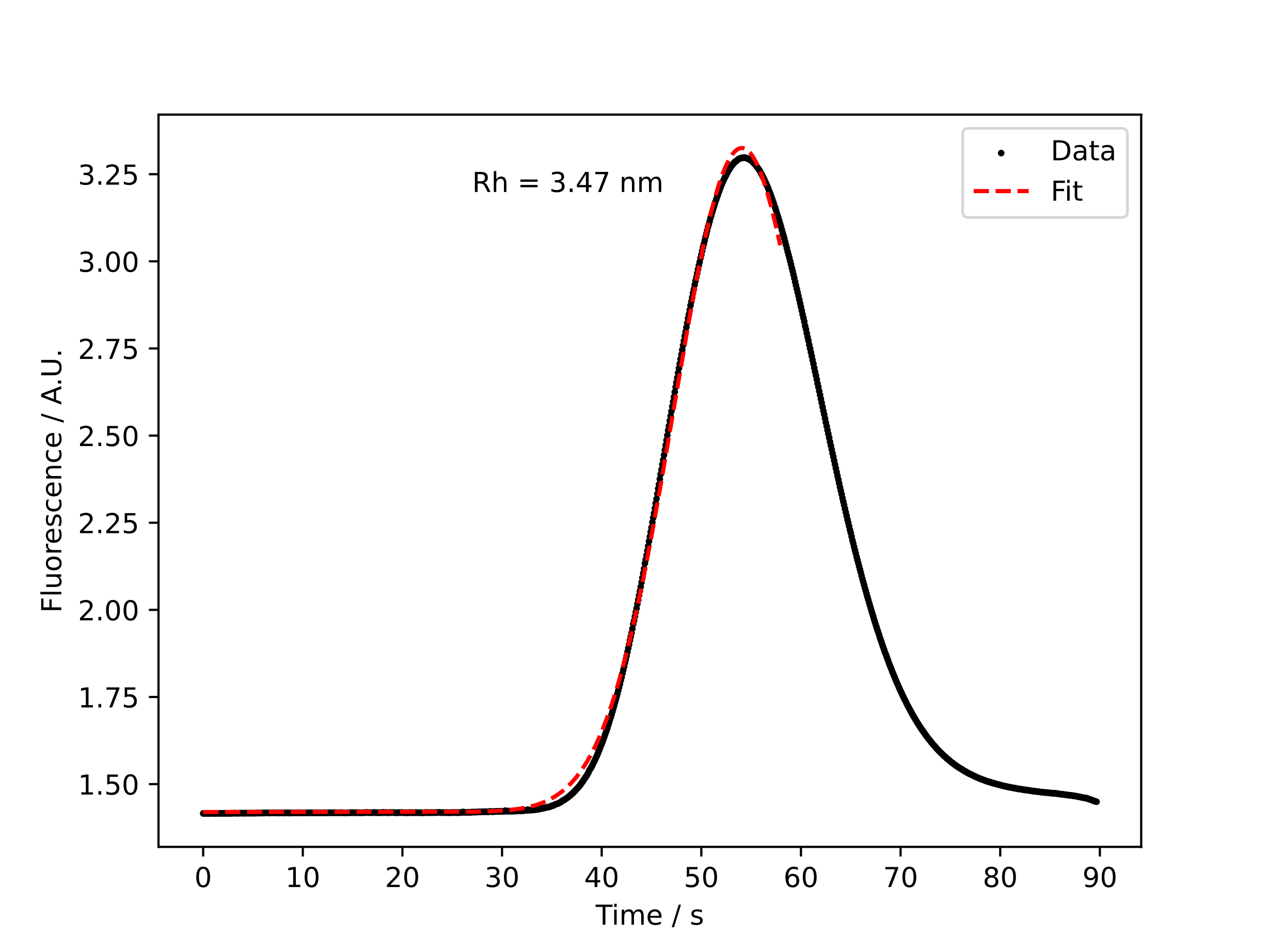


Fig 3S: Taylor-gram of sE Asibi protein (1.89 mg/mL) after 4 weeks storage at -80 °C. Measurements were performed on the Fida 1 instrument (Fida biosystems, Denmark). Detection is based on intrinsic tryptophane fluorescence of the protein and measured with the Fida Neo UV detector with a 280 nm LED for excitation and a 375 nm long pass filter for emission (Fida biosystems, Denmark). A permanently coated (LPA) capillary of 1 m length with a 75 µm diameter (Fida biosystems, Denmark) was used and samples were injected from the compatible V-bottom 96 well plate and from the standard 50-vial holder. Two replicates of YFV sE-Asibi protein were performed. The Taylor-grams acquired by the FIDA measurements were analyzed using the Fida Software (V3.1.2.0).

Fig. 4S: BLI measurement of sE-Asibi


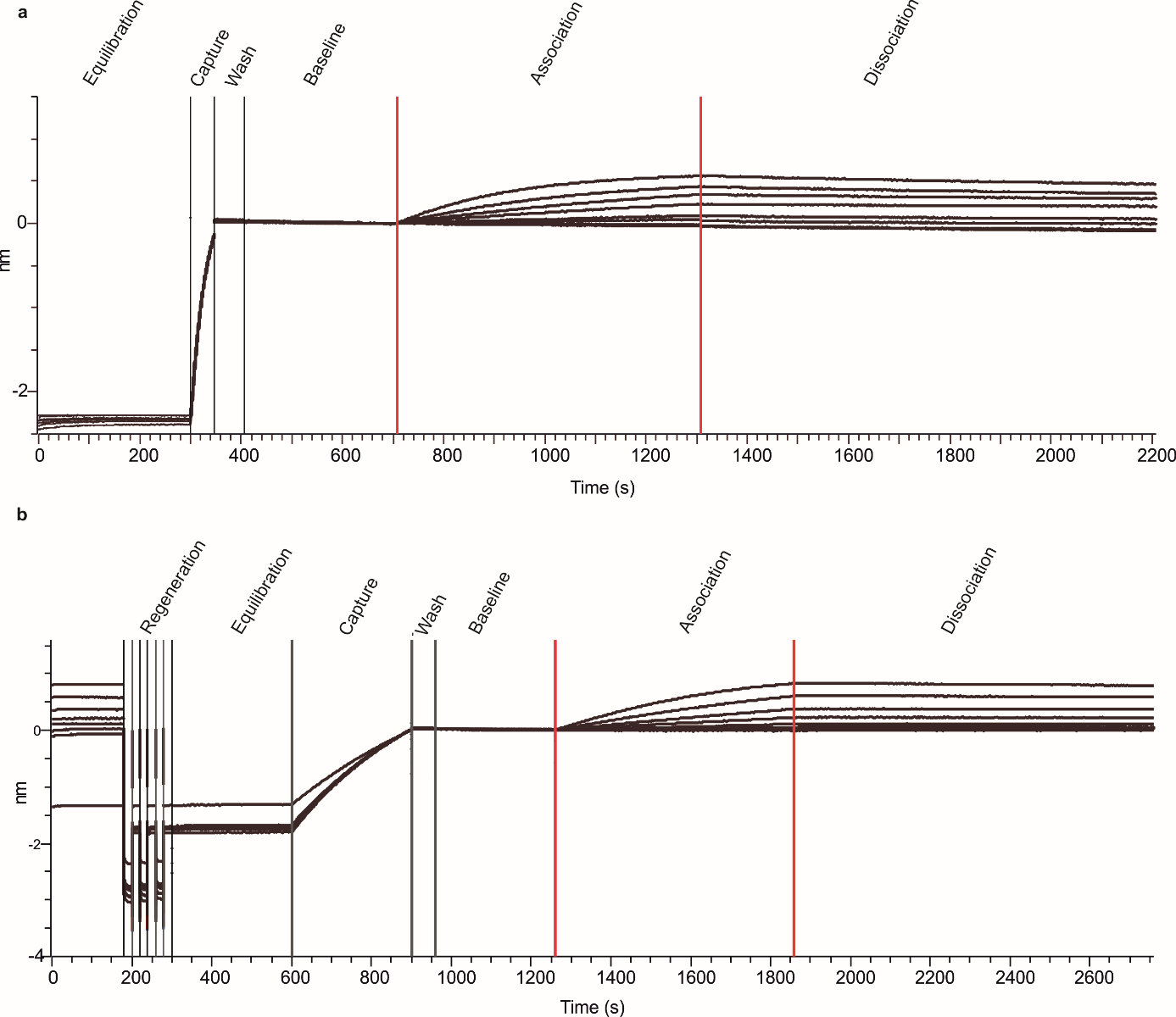


Fig. 4S: Description of the BLI steps performed on a) the AMC2 biosensors with the mouse antibody D1-AG2-4-15 captured and b) AHC2 biosensors with the human antibody 5A-IgG1 captured. The capture of the antibody was always preceded by an equilibration step for 300 sec. Association was recorded for 600 sec and dissociation for 900 sec. A preliminary experiment was performed with the two different sensors to identify the best capture conditions giving rise to optimal kinetic experiments at a given concentration of sE protein.

Fig. 5S: Tryptic peptide mass fingerprint coverage of the sE-17D construct

MGTLVRKNRWLLLNVTSEDLGKTFSVGTGNCTTNILEAKYWCPDSMEYNCPNLSPREEPDDIDCWCYGVENVRVAYGKCDSAGRSRRSRRAIDLPTHENHGLKTRQEKWMTGRMGERQLQKIERWFVRNPFFAVTALTIAYLVGSNMTQRVVIALLVLAVGPAYSAHCIGITDRDFIEGVHGGTWVSATLEQDKCVTVMAPDKPSLDISLETVAIDRPAEVRKVCYNAVLTHVKINDKCPSTGEAHLAEENEGDNACKRTYSDRGWGNGCGLFGKGSIVACAKFTCAKSMSLFEVDQTKIQYVIRAQLHVGAKQENWNTDIKTLKFDALSGSQEVEFIGYGKATLECQVQTAVDFGNSYIAEMETESWIVDRQWAQDLTLPWQSGSGGVWREMHHLVEFEPPHAATIRVLALGNQEGSLKTALTGAMRVTKDTNDNNLYKLHGGHVSCRVKLSALTLKGTSYKICTDKMFFVKNPTDTGHGTVVMQVKVSKGAPCRIPVIVADDLTAAINKGILVTVNPIASTNDDEVLIEVNPPFGDSYIIVGRGDSRLTYQWHKEGSSGGSGGSLEVLFQGPSAWSHPQFEKGGGSGGGSGGSAWSHPQFEK

Fig. 5S: Peptide coverage after deglycosylation and tryptic digest of the purified fusion protein 17D prM-E-3C-TST; peptides identified with high significance are highlighted by grey boxes. Grey boxes framed by a dotted line highlight peptides that were additionally identified by in-gel tryptic digest of the SDS-PAGE separated peptide band (main manuscript Fig. 3 a).

Fig. 6S: Intact mass measurement of sE-Asibi after deglycosylation


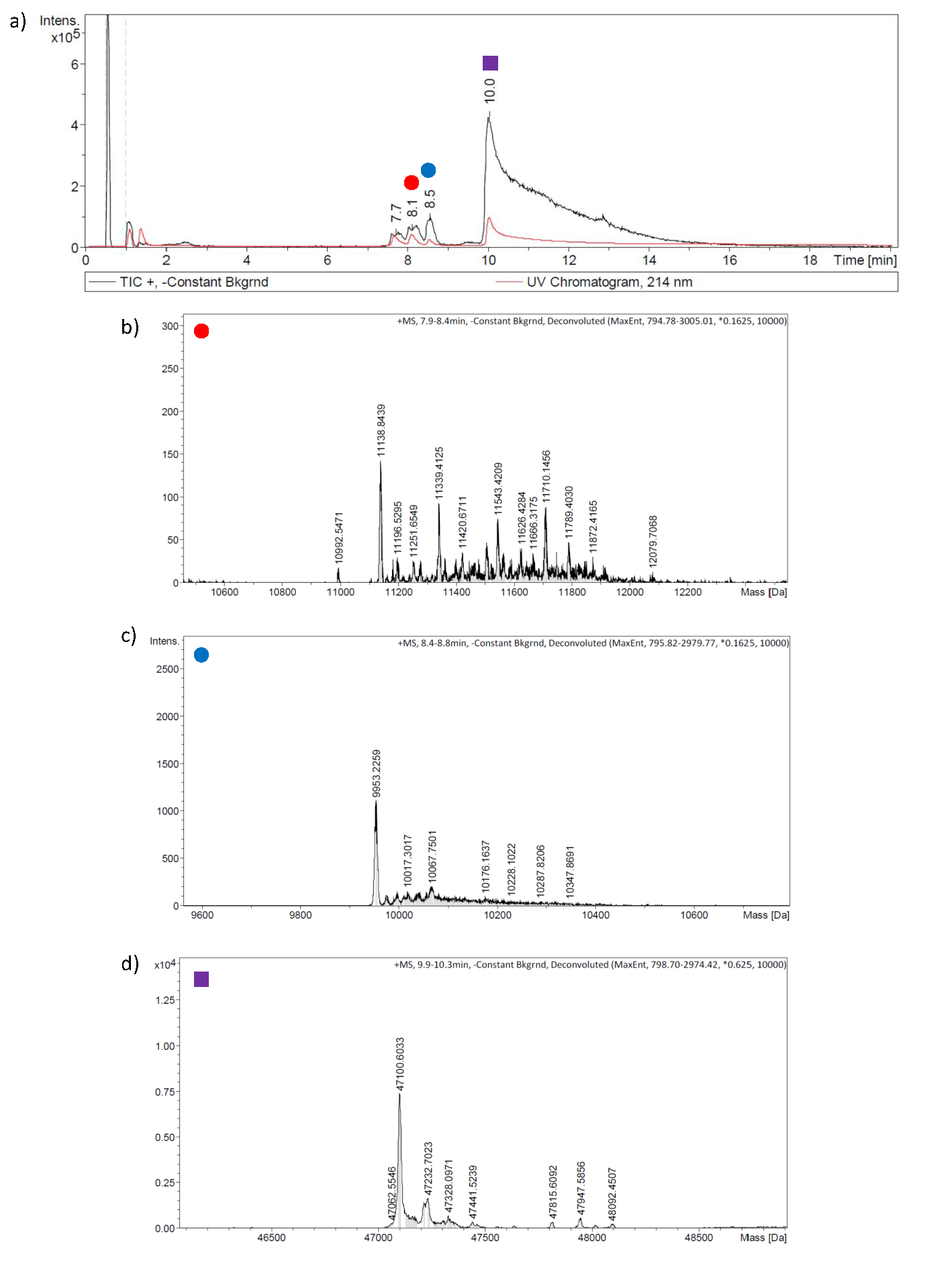


Fig. 6S: Chromatogram of HPLC separation of sE-Asibi protein with total ion count (TIC) indicated in black and UV absorption at 214 nm wavelength indicated in red (a). Four significant peaks were detected. The 7.7 min peak corresponds to non-deglycosylated protein for which only a diffuse size distribution around 12 kDa, but no accurate molecular mass could be derived (data not shown); b), c) and d) masses identified for the HPLC elution peaks at 8.1 min, 8.5 min and 10 min, respectively, listed in Table 1.

Fig. 7S: Native mass spectrometry analysis of sE-Asibi deglycosylated


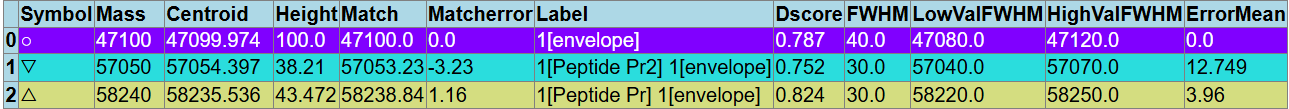


1. b)


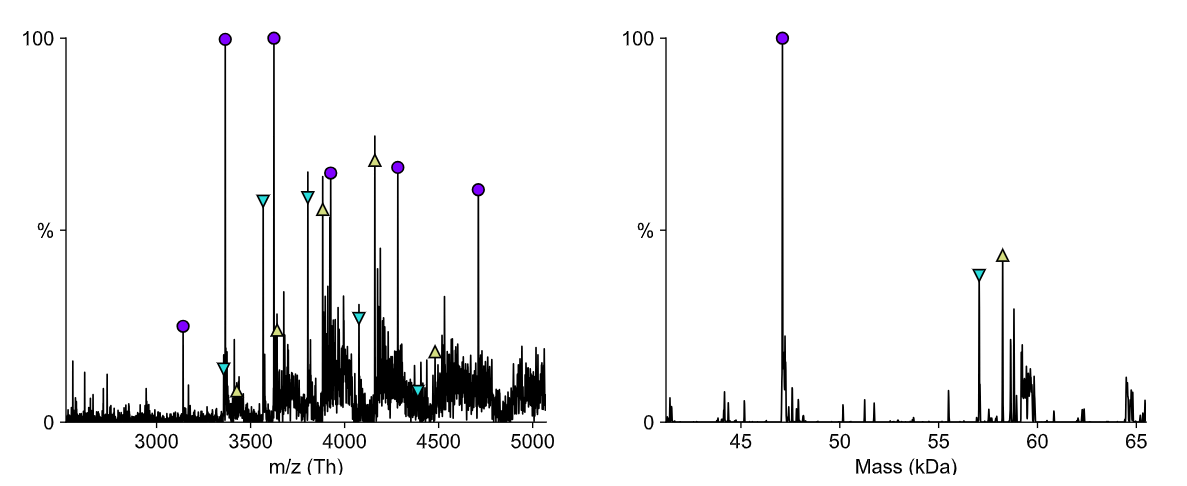


Fig. 7S: a) raw native mass spectrum of deglycosylated sE-Asibi showing different m/z for envelope (violet), pr1 peptide bound to envelope (blue) and pr2 peptide bound to envelope (yellow-green); b) deconvoluted mass spectrum showing the individual masses of envelope, pr1 peptide bound to envelope and pr2 peptide bound to envelope in kDa.

Fig. 8S: original, uncropped images of Fig. 2 a, b and c of the main manuscript


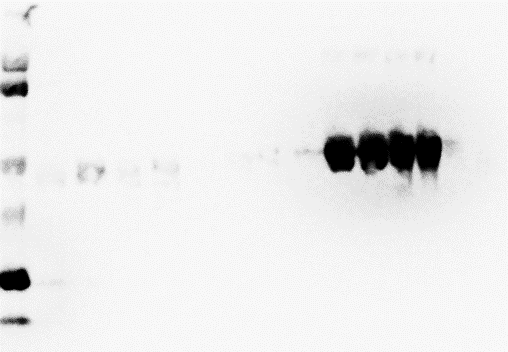

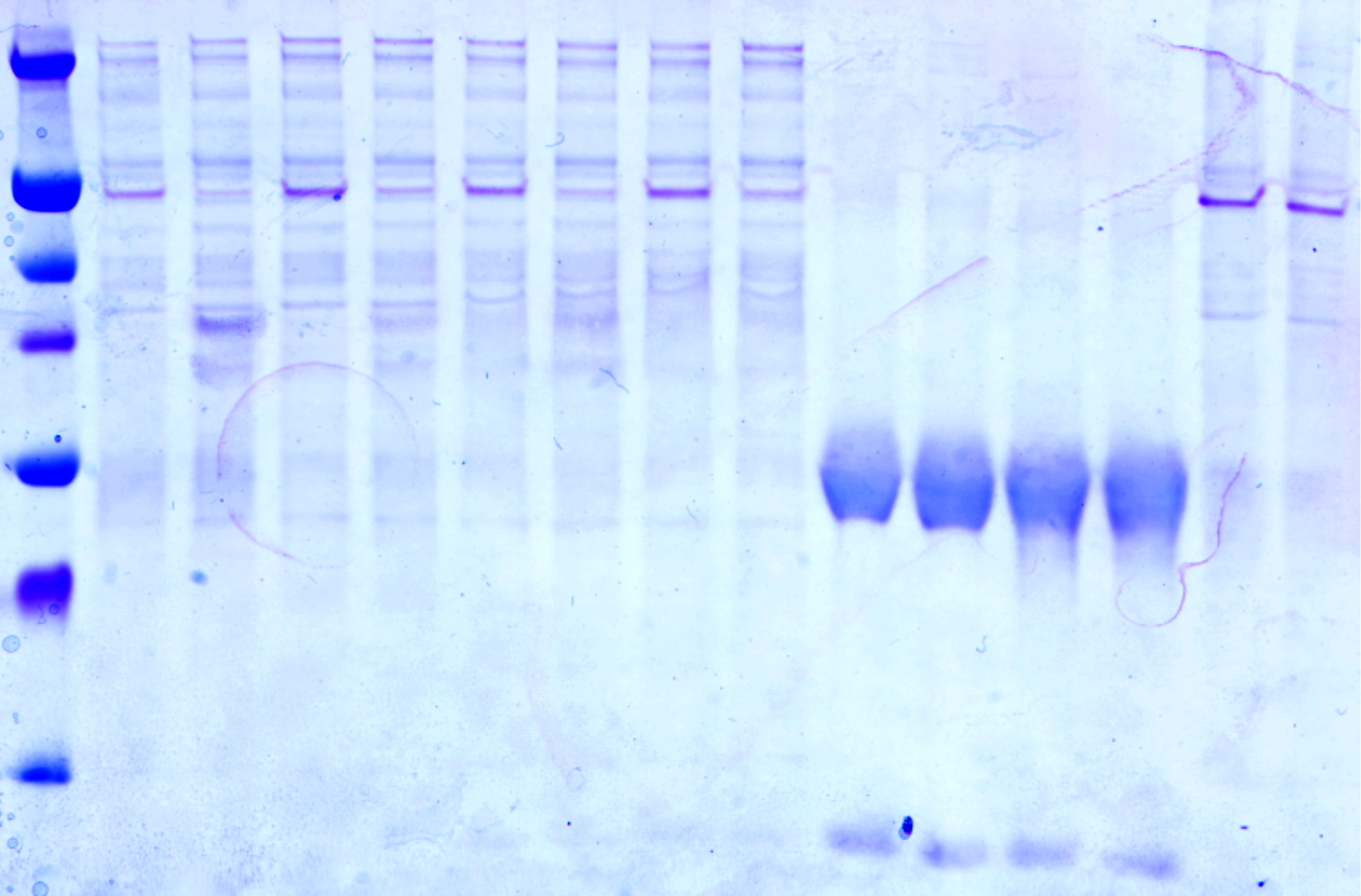

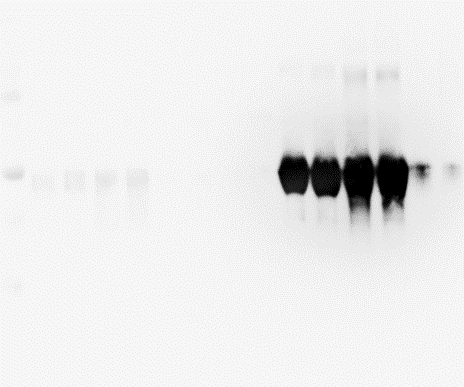


**a**

**b**

**c**

**Anti-TST**

**Anti-E**

**Coomassie**

Fig. 8S: original, uncropped images of Fig. 3 a, b and c of the main manuscript


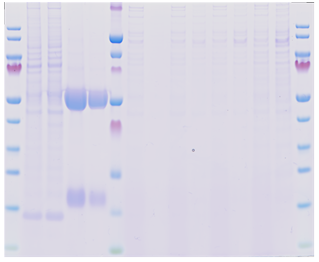

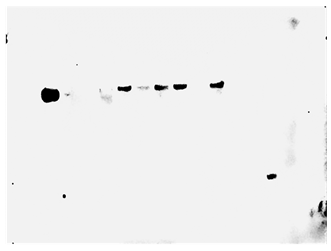

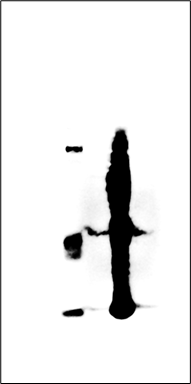


**c**

**a**

**Anti-E**

**b**

**Anti-prM**

**Coomassie**
